# Deregulation of DNA Double-Strand Break Repair in Multiple Myeloma: Implications for Genome Stability

**DOI:** 10.1371/journal.pone.0121581

**Published:** 2015-03-19

**Authors:** Ana B. Herrero, Jesús San Miguel, Norma C. Gutierrez

**Affiliations:** 1 Servicio de Hematología, Hospital Universitario, IBSAL, IBMCC (USAL-CSIC), Salamanca, Spain; 2 Clínica Universidad de Navarra, Centro de Investigaciones Médicas Aplicadas (CIMA), Pamplona, Spain; Institut Pasteur, FRANCE

## Abstract

Multiple myeloma (MM) is a hematological malignancy characterized by frequent chromosome abnormalities. However, the molecular basis for this genome instability remains unknown. Since both impaired and hyperactive double strand break (DSB) repair pathways can result in DNA rearrangements, we investigated the functionality of DSB repair in MM cells. Repair kinetics of ionizing-radiation (IR)-induced DSBs was similar in MM and normal control lymphoblastoid cell lines, as revealed by the comet assay. However, four out of seven MM cell lines analyzed exhibited a subset of persistent DSBs, marked by γ-H2AX and Rad51 foci that elicited a prolonged G2/M DNA damage checkpoint activation and hypersensitivity to IR, especially in the presence of checkpoint inhibitors. An analysis of the proteins involved in DSB repair in MM cells revealed upregulation of DNA-PKcs, Artemis and XRCC4, that participate in non-homologous end joining (NHEJ), and Rad51, involved in homologous recombination (HR). Accordingly, activity of both NHEJ and HR were elevated in MM cells compared to controls, as determined by *in vivo* functional assays. Interestingly, levels of proteins involved in a highly mutagenic, translocation-promoting, alternative NHEJ subpathway (Alt-NHEJ) were also increased in all MM cell lines, with the Alt-NHEJ protein DNA ligase IIIα, also overexpressed in several plasma cell samples isolated from MM patients. Overactivation of the Alt-NHEJ pathway was revealed in MM cells by larger deletions and higher sequence microhomology at repair junctions, which were reduced by chemical inhibition of the pathway. Taken together, our results uncover a deregulated DSB repair in MM that might underlie the characteristic genome instability of the disease, and could be therapeutically exploited.

## Introduction

Multiple myeloma (MM) is a clonal disorder of B-cells at the last stage of differentiation. Genome instability is a prominent feature of MM cells, and includes ploidy changes, deletions, amplifications and chromosomal translocations mainly involving the *IGH* locus on chromosome 14q32 [1]. However, the underlying molecular mechanisms for the generation of this instability are unclear. Numerical chromosome abnormalities may be generated by centrosome amplification or alterations in the spindle assembly checkpoint (SAC) [2,3]. On the other hand, structural abnormalities, such as chromosomal deletions or translocations, might arise from alterations in the repairing of DNA double strand breaks (DSBs).

DSBs can be generated exogenously, by the exposure to a variety of genotoxic agents, or endogenously, during normal cellular processes such as DNA replication, or lymphoid V(D)J and class-switch recombination (CSR), occurring at the *IGH* locus on chromosome 14q32 [4]. One of the first responses to the presence of a DSB is the phosphorylation of histone H2AX by members of the PI3-K family, such as ataxia telangiectasia mutated (ATM), ataxia telangiectasia and Rad3 related (ATR) or DNA-dependent protein kinase catalytic subunit (DNA-PKcs) [4,5]. Once damage is detected, DSBs can be repaired by two major pathways: homologous recombination (HR) and non-homologous end joining (NHEJ) [6,7,8]. During HR, the sister chromatid is used as a template to copy the missing information into the broken locus. In contrast, NHEJ proceeds by a direct ligation of the two broken ends, and can produce short deletions or insertions [7]. The pathway is initiated at the sites of DSBs by the Ku70/Ku86 heterodimer that binds the broken DNA ends, and recruits the DNA-PKcs. The DNA-PK complex stabilizes the DNA ends and a ligation reaction is then carried out by the DNA ligase IV/XRCC4 complex. The role of HR and NHEJ in cancer is complex since both underactivity and overactivity can contribute to genome instability and to the development or progression of the disease [9,10,11,12].

Recent results have shown the existence of an alternative, and still poorly defined end joining pathway (Alt-NHEJ), that is mainly operative when the classical NHEJ pathway is impaired [13,14]. Alt-NHEJ requires more extensive end resection, and frequently uses microhomology in the repair. Moreover, it has been implicated in the chromosomal translocations that give rise to lymphoid cancers [14,15,16,17].

Here, we investigated the functionality of DSB repair in MM by different approaches. Our results showed that several MM cell lines accumulate a subset of persistent DSBs after irradiation that makes them hypersensitive to IR and dependent on a functional G2/M checkpoint for survival. However, NHEJ, HR and Alt-NHEJ repair pathways are upregulated in MM cells probably contributing to the repair of endogenous DNA damage, but increasing genome instability, which may result in disease progression and acquisition of drug resistances.

## Materials and Methods

### Ethics statement

The use of clinical samples for investigation was approved by the Ethical Committee of the University Hospital of Salamanca and patients gave their written consent for that use.

### Cells and culture conditions

The human myeloma cell lines, NCI-H929 and MM1S were acquired from ATCC (American Type Culture Collection) and JJN3, RPMI-8226, U266, OPM2 and IM9, from DMSZ (Deuthche Sammlung von Mikroorganismen and Zellkulturen). LINF167, LINF692 and LINF903, Epstein-Barr virus (EBV)–transformed B-cell lines established from 3 healthy individuals, were obtained from the National DNA Bank of the University of Salamanca (Spain). MM and LINF cell lines were cultured in RPMI 1640-L-Glutamine medium (Sigma-Aldrich, St Louis, MO) supplemented with 10% of fetal bovine serum (FBS) (Sigma-Aldrich) and antibiotics (Gibco Life Technologies, Grand Island, NY). HeLa and HCT116 were obtained from the ATCC and were cultured in DMEM supplemented with L-glutamine (Sigma-Aldrich), 10% FBS and antibiotics. All cells were incubated at 37°C in a 5% CO_2_ atmosphere. The presence of mycoplasma was routinely checked with MycoAlert kit (Lonza, Basel, Switzerland) and only mycoplasma free cells were used in the experiments. Bone marrow (BM) samples were obtained from 5 patients with MM with written informed consent in accordance with the Declaration of Helsinki.

### Cell irradiation

Cells were irradiated during the exponential phase of cell growth with γ-rays using a Gammacell 1000 Elite irradiator (Cesium^137^), at a dose rate of 243 cGy/min. Samples were collected at the indicated times after irradiation and processed for flow cytometry or inmunofluorescence staining.

### Flow cytometry analysis of γH2AX

Cells were fixed in 1ml 70% ethanol, rehydrated in phosphate buffer saline (PBS) and permeabilized on ice by incubation for 15 min in PBS containing 0.25% Triton X-100. Antibody staining was performed by incubation for 2h with anti-phospho-Histone H2AX (Ser-139) antibody (mouse, Merck Millipore, Darmstadt, Germany) diluted 1:1,000 in 100 μl of 1% BSA in PBS. Cells were washed and resuspended in secondary antibody (Alexa Fluor 488 goat anti-mouse IgG (H+L), Invitrogen, Carlsbad, CA) diluted 1:1,000 in 1% BSA in PBS and incubated 1h at RT. A Becton Dickinson (Mountain View, CA) FACSCalibur was used to measure γH2AX and DNA content after washing and resuspending cells in 500 μl of PI/RNase staining solution (Immunostep, Salamanca, Spain).

### Inmunofluorescence staining

Cells (50,000) were mounted onto glass slides by cytospinning for 10 min at 1,000 rpm. Cells were fixed in 2% paraformaldehyde for 20 min, permeabilized in 0.2% Triton X-100 in PBS for 10 min, blocked in 3% BSA in PBS for 30 min and incubated with anti–γ-H2AX (Millipore) or anti-Rad51 (mouse, Santa Cruz Biotechnology, Santa Cruz, CA) at 1:1,000 dilution for 2h. After washing, slides were incubated with fluorescent secondary antibodies (1:1,000, Alexa Fluor 488 goat anti-mouse IgG or Alexa Fluor 568 anti-rabbit) for 1h. Slides were mounted with ProLong Gold antifade reagent (Invitrogen) and images were acquired using a DeltaVision system made up of a Olympus IX71 microscope, a Photometrics Coolsnap camera and sofWoRx software. A 60X oil immersion objective was used.

### Comet assay

Cells were treated with the apoptosis inhibitor Z-VAD-FMK (CliniSciences, Barcelona, Spain) at 30 μM, and then irradiated with 40 Gy of IR. Samples were collected at different time points and processed for neutral comet assay as described previously [18] with some modifications. Briefly, cell density were adjusted to 10^5^ cells/ml in ice-cold PBS and mixed with LMAgarose (Trevigen, Gaithersburg, MD), at 37°C, at a ratio of 1: 10 (v/v). Cell suspensions (25 μl) were immediately transferred onto CometSlide slides (20 well slides) and placed at 4°C for 10 minutes. Slides were submerged in N1 lysis solution [18] and incubated overnight at 37°C in the dark. After rinsing in N2 buffer [18], slides were subjected to electrophoresis in N2 solution for 30 min at 1V/cm. Cells were stained with ethidium bromide and analyzed with a fluorescence microscope (Zeiss Axioplan 2) equipped with a Hamamatsu Orca-EC camera. Images were obtained using Openlab software. At least 100 images per sample and experiment were analyzed. Tail moment was determined by the OpenComet software [19].

### Cell cycle analysis

Cells were washed in PBS and fixed in 70% ethanol for later use. Cells were rehydrated with PBS, resuspended in 500 μl of PI/RNase staining solution (Immunostep), and incubated for 20 minutes at RT in the dark. Samples were analyzed using a FACSCalibur flow cytometer.

### Cell apoptosis assays

Apoptosis was measured using an annexin V-fluorescein isothiocyanate/propidium iodide (PI) double staining (Immunostep, Spain) according to the manufacturer's procedure.

### Immunoblotting

Cells were washed with PBS and lysed in ice-cold lysis buffer (140 mmol/l NaCl, 50 mmol/l EDTA, 10% glycerol, 1% Nonidet P-40, 20 mmol/l Tris HCl pH 7) containing protease inhibitors (Complete, Roche Applied Science, Indianapolis). Protein concentration was measured using the Bradford assay (BioRad, Hercules, CA). Protein samples (20 μg/lane) were subjected to SDS-PAGE and transferred to PVDF membrane (BioRad). After blocking, membranes were incubated with anti-human antibodies. The following primary antibodies were used: anti-Ku70 (A-9, mouse, Santa Cruz Biotechnology), anti-Ku86 (S10B1, mouse, Santa Cruz Biotechnology), anti DNA-PKcs (rabbit, Abcam, Cambridge, UK), anti-DNA ligase IV (rabbit, Abcam), Anti-Artemis (goat, Abcam), anti-XRCC4 (A-7, mouse, Santa Cruz Biotechnology), anti-DNA ligase IIIα (1F1, mouse, Gene Tex, Irvine, CA, USA), anti-WRN (H-300, rabbit, Santa Cruz Biotechnology), anti-Rad51 (Rabbit, Santa Cruz Biotechnology). Horseradish peroxidase–linked donkey anti-rabbit, anti-mouse or anti-goat antibodies (Santa Cruz Biotechnology) were used as secondary antibodies at 1:5,000 dilution. Immunoblots were incubated for 1h at RT and developed using enhanced chemiluminescence western blotting detection reagents (Amersham Biosciences, Piscataway, NJ).

### NHEJ assays

The end joining reporter plasmid pEGFP-Pem1-Ad2 was used to determine the in vivo levels of NHEJ [20]. Digestion with *HindIII* or *I-SceI* enzymes eliminates the Ad2 sequence within Pem1 intron and generates compatible or incompatible ends, respectively. EGFP signal can be recovered if the transfected cells possess end joining activity to recircularize the linear plasmids. pDSRed2-N1 plasmid (Clontech, Palo Alto, CA, USA) was cotransfected with either linearized pEGFP-Pem1-Ad2 or supercoiled pEGFP-Pem1 (obtained by religation of Hind*III*-digested pEGFP-Pem1-Ad2) to evaluate the transfection efficiency. Red-versus green curves were generated for the different cell lines with varying amounts of red and green plasmids to avoid measurements near the plateau region. Higher numbers of GFP+ compared to DsRed+ cells were obtained even when increased amounts of red *vs* GFP plasmid were assayed, as previously described [21]. We fixed an amount of 2 μg of pDSRed2-N1 and 0.5 μg of either linearized pEGFP-Pem1-Ad2 or supercoiled pEGFP-Pem1 for the experiments. One million cells were transfected using the Amaxa Cell Line Nucleofector Kit V and Amaxa Nucleofector device (Lonza, Allendale, NJ, USA). Programs used were X-005 for U266 cell line, T-016 for H929 and JJN3, S-020 for MM1S, and G-016 for RPMI-8226. Green (EGFP) and Red (DsRed) fluorescence were measured 24h later using a BD Accuri C6 flow cytometer (Franklin Lakes, NJ, USA). A total of 200,000 cells per sample were analyzed. NHEJ efficiency was calculated by dividing the number of EGFP positive cells arising from circularized linear plasmid by the number of transformants arising from parallel transfections of undigested plasmid DNA, after normalizing from transfection efficiency, X 100.

### Construction of cell lines for detecting NHEJ efficiency

U266, JJN3, LINF692 and LINF903 were transfected with 1 μg of the NHEJ-C reporter construct linearized by digestion with *Nhe*I [22]. G418 was added at 500 μg/ml 3 days post-transfection and stable pools were obtained after 3 weeks of selection, in the case of U266 and JJN3, or 2 months for LINF cell lines. Medium containing G418 was changed every 3 days. To measure NHEJ efficiency in stable pools, cells were transfected with 5 μg of plasmid encoding I-SceI endonuclease and 2 μg of pDSRed2-N1. NHEJ efficiency was calculated 24h later as the ratio of GFP+/DsRed+ cells.

### Repair fidelity assay

*EcoR*I-linearized pUC18 plasmids were transfected into MM cell lines using the programs and conditions detailed above. Successful repair results in re-circularization of the plasmid with restoration of β-galactosidase activity. Plasmid DNA was extracted from the cells 24h post-transfection [QIAprep spin miniprep kit (Qiagen, Germany)], and transformed into *E*. *coli* DH5α cells. After plating on agar plates containing IPTG and X-Gal (Sigma-Aldrich), numbers of white and blue colonies were counted. The nature of misrepair was analyzed in plasmids obtained from white colonies by PCR and sequencing of the breakpoint junction. The primers used were: pUC18-5, cggcatcagagcagattgta, and pUC18-3, tggataaccgtattaccgcc.

### HR assays

The HR reporter plasmid was used to determine the in vivo levels of HR [22]. The plasmid was digested with the restriction enzyme *Sce*I and purified. To evaluate the transfection efficiency 2 **μ**g of the HR construct, together with 2 **μ**g of pDsRed-N1, were cotransfected into the cells using the conditions and programs detailed for the NHEJ assays. GFP+ and DsRed+ were quantified by flow cytometry 48h after transfection. One million events per sample were analyzed. Efficiency of HR was calculated by dividing the number of GFP+ cells arising from the linear plasmid by the number of DsRed+ cells.

### Statistic

Differences between the data were assessed for statistical significance using the Student's unpaired two tailed *t*-test with the Simfit statistical software version 7.0.5 (http://www.simfit.org.uk/).

## Results

### Several MM cell lines exhibit persistent DSBs and a strong G2/M checkpoint response after irradiation

**Fig 1:**
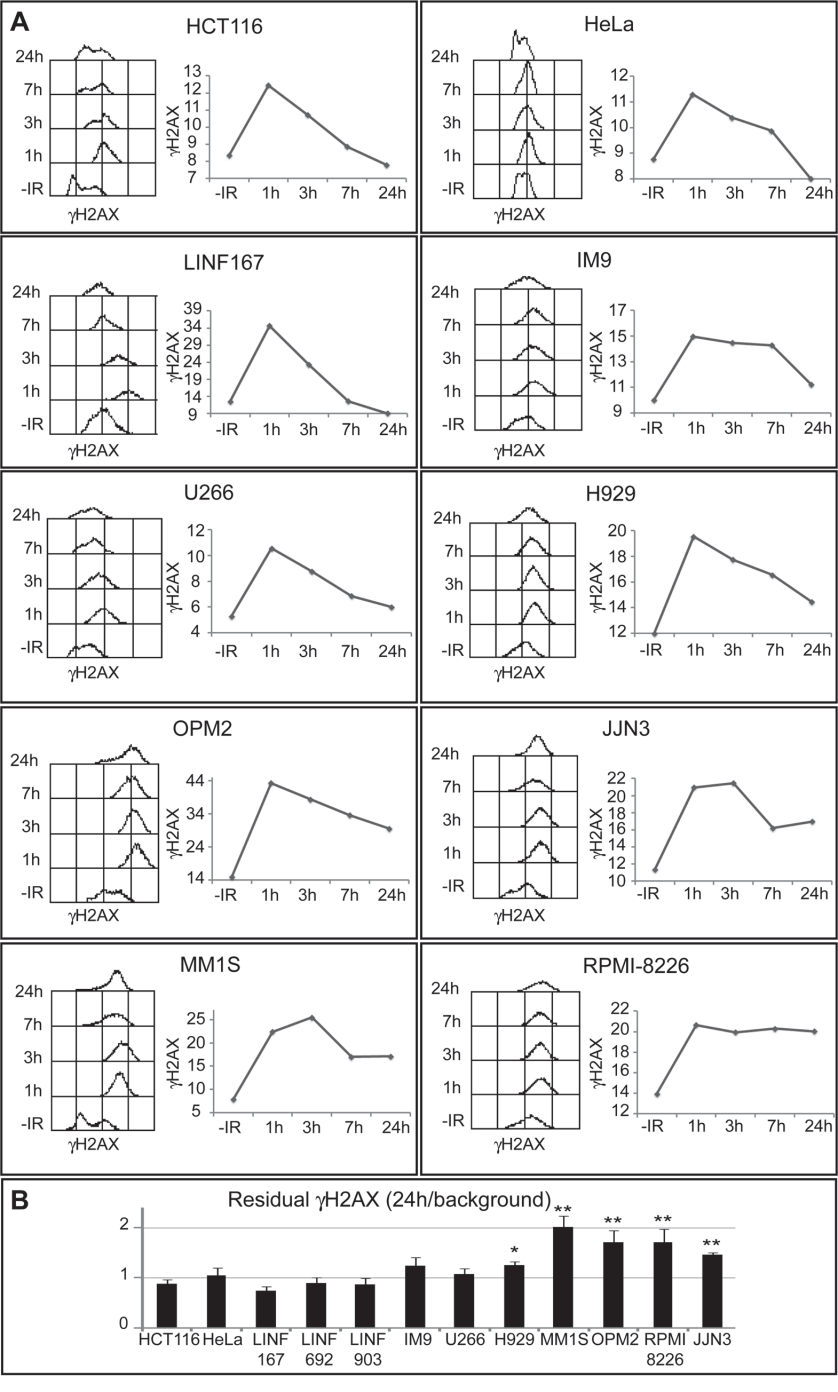
Kinetics of γH2AX loss following IR. (A) Asynchronous cells were treated with 2 Gy IR, fixed at the indicated times post-irradiation, and stained with anti- γH2AX and secondary fluorescent antibodies. Kinetics of γH2AX disappearance is illustrated for each cell line by a histogram, showing the levels of γH2AX at different times post-IR, and a graphic, where the mean intensity of γH2AX (in arbitrary units) is plotted. Best representative from several independent experiments is shown. Similar results were obtained for all LINF cell lines (only LINF167 is shown). (B) Residual γH2AX, quantified as the ratio of the signal at 24h post-IR/signal in non-irradiated cells, was obtained as the mean of three independent experiments. Error bars correspond to standard deviation (SD) (** p<0.01, * p<0.05, compared to LINF cells, Student's *t*-test).

To analyze DSB formation and repair we first monitored the phosphorylation of H2AX (γH2AX), a sensitive marker of DSBs [23], after treatment with 2 Gy of ionizing radiation (IR). γH2AX signal was quantified by flow cytometry in 7 MM cells lines and compared to 5 cell lines (3 lymphoblastoid cell lines obtained from normal lymphocytes, HeLa and HCT116), that were used as repair-proficient controls (Fig. 1A). We found that γH2AX intensity reached its maximum at 1h post-IR in most of the cell lines analyzed, and started to fall over the next 24h. However, whereas γH2AX signal decreased with a fast kinetics in controls and U266 cells, and with an intermediate kinetics in IM9 and H929 cells, the reduction of γH2AX was slower in OPM2, JJN3, MM1S and especially in RPMI-8226, which suggests a defect in DSB repair at least in these 4 MM cell lines. Residual γH2AX, quantified as the ratio of the signal at 24h post-IR/signal in non-irradiated cells, showed significantly higher values in OPM2, JJN3, MM1S and RPMI-8226 than in LINF control cell lines (Fig. 1B).

**Fig 2:**
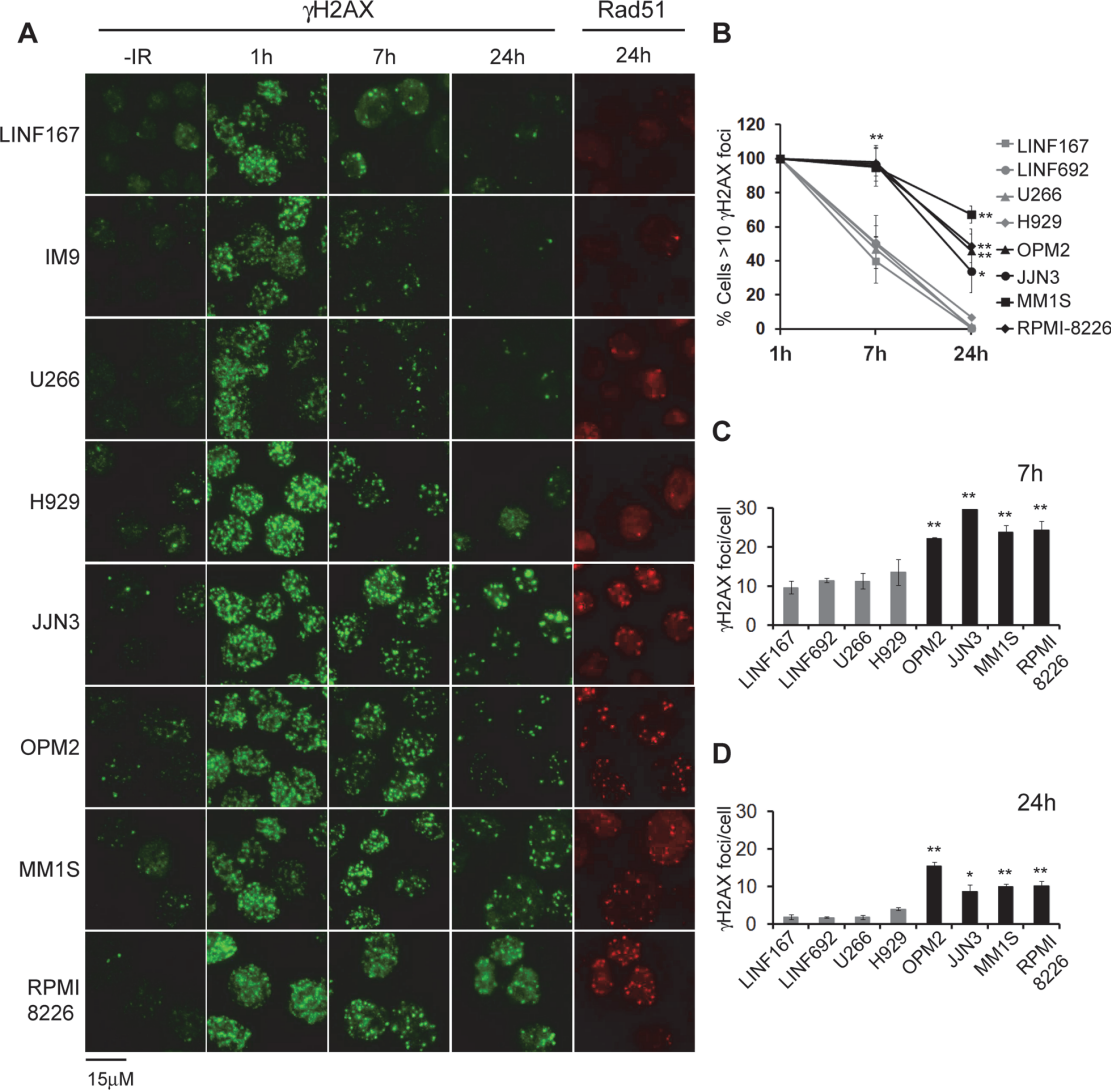
γH2AX foci after exposure to IR (2 Gy). (A) Representative images of γH2AX foci in untreated cells, and in cells irradiated with 2 Gy 1h, 7h and 24h post-IR. Rad51 foci in cells at 24h post-IR are also shown. (B) Percentage of cells with γH2AX foci at the indicated times post-IR. (C) and (D) Quantification of the number of γH2AX foci per cell at 7 and 24h post-IR. In all quantifications data represent the mean values of at least 2 independent experiments. (** p<0.01, * p<0.05, compared to LINF cells). A minimum of 100 cells per experiment and cell line were counted.

**Fig 3:**
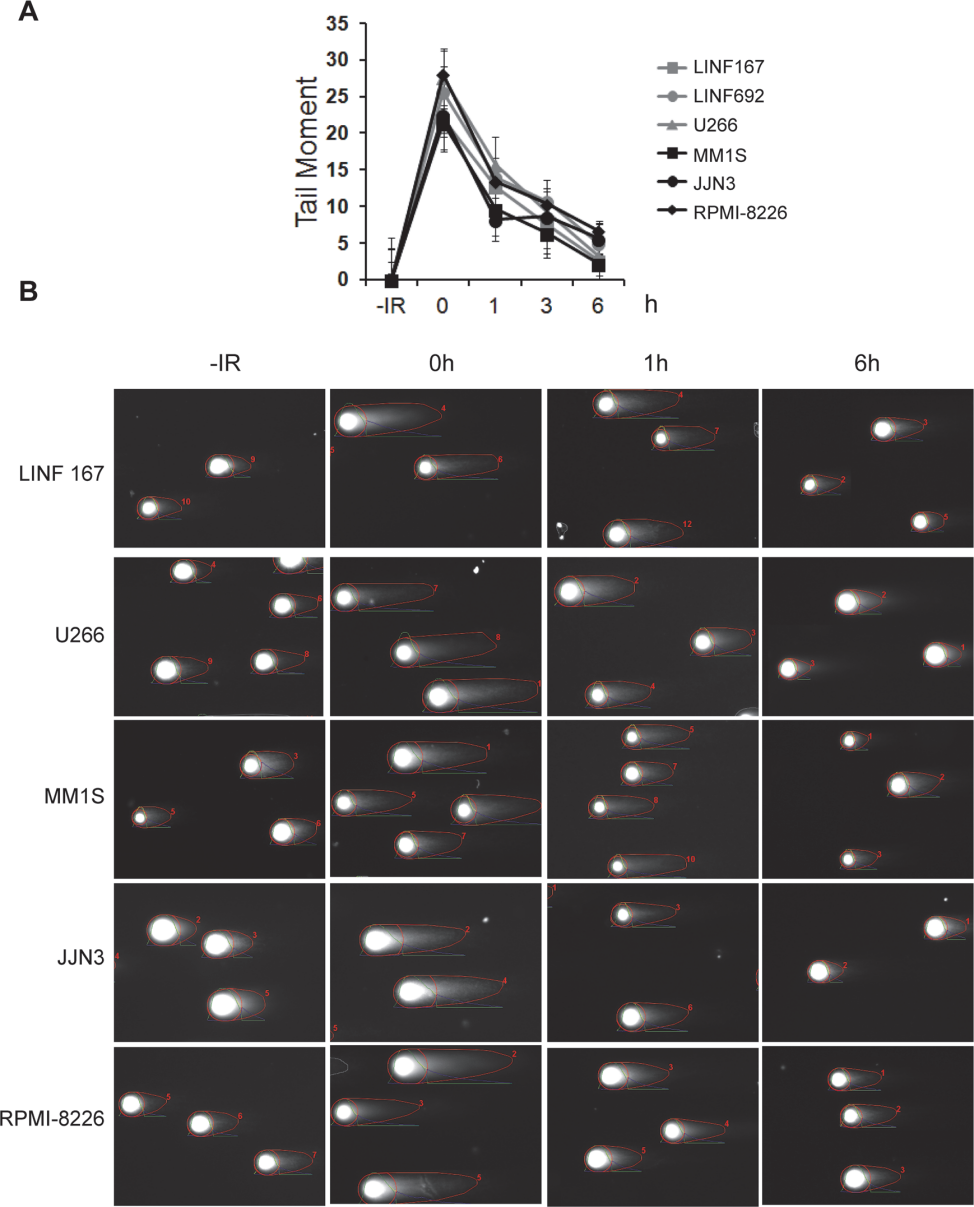
Analysis of DSB repair by the neutral comet assay. (A) Cells were irradiated with 40 Gy of IR and mean tail moment calculated at different time points using the OpenComet software. Data represent mean values, after subtraction of background damage, of three independent experiments ± SD. At least 100 cells were scored per sample and experiment. (B) Representative images of one experiment.

To corroborate the results obtained by flow cytometry, we analyzed γH2AX foci at different times post-IR (2 Gy). In the absence of treatment, all MM cell lines, with the exception of IM9 and U266, exhibited more γH2AX foci than controls (Fig. 2A), in agreement with a previous report that described increased endogenous DNA damage in MM cells [24]. We found that OPM2, JJN3, MM1S, and RPMI-8226 were able to repair many IR-induced breaks, since foci numbers decreased from 1h to 7h and 24h post-IR (Fig. 2A). Even so, the percentage of cells with γH2AX foci (Fig. 2B) and the number of foci per cell at 7h and 24h post-IR (Fig. 2C and 2D) was higher in those cells lines than in U266, H929 or LINF control cells, which corroborated the results previously obtained by flow cytometry. We observed that most of the γH2AX foci present at 24h post-IR colocalized with Rad51 foci (Fig. 2A). Next, we analyzed the repair kinetics of IR-induced DSBs using the neutral comet assay. Surprisingly, no statistically significant differences were found in the kinetics of DSB repair between MM and control LINF cell lines (Fig. 3). In all the cases most of the DNA damage seemed to be repaired 6h after irradiation, despite the high irradiation dose applied (40 Gy), in agreement with results previously described in other non myeloma cell lines [25]. These results indicate that MM cells are able to repair most of the IR-induced DSBs, while the subset of persistent DSBs in OPM2, JJN3, MM1S and RPMI-8226, identified as γH2AX foci, might correspond to lesions, especially difficult to repair in these MM cell lines, and below the comet assay detection limit (on the order of 50–75 breaks per cell, as previously described [25]).

**Fig 4:**
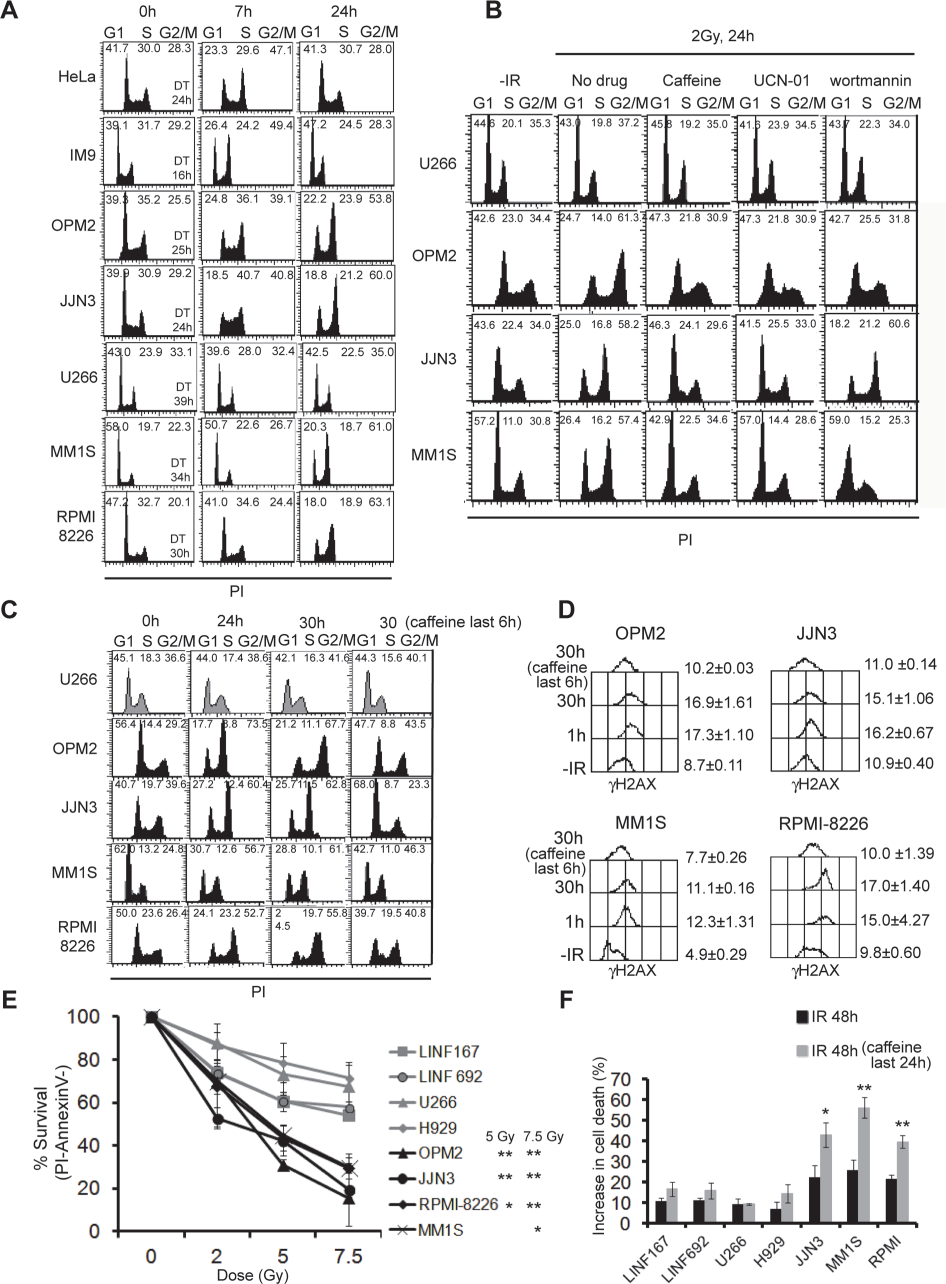
Cell cycle phase distribution and cell survival after exposure to IR. (A) Cell cycle analysis. Cells were fixed at the indicated times post-IR and DNA content was measured by flow cytometry. Percentages of cells in the different phases of the cell cycle are indicated and duplication times (DT) were calculated (http://www.doubling-time.com/compute.php). Best representative from several independent experiments is shown. (B) Cell cycle distribution of cells at the indicated times post-IR (2 Gy) in the presence or in the absence of the checkpoint inhibitors (caffeine, UCN-01 and wortmannin). (C) Caffeine (4 mM) was added 24h post-IR and maintained for 6h. (D) Levels of γH2AX (in arbitrary units) in the absence of treatment (-IR), 1h, 30h post-IR and 30h post-IR with the last 6h in the presence of caffeine. Data are the mean of two independent experiments. (E) Cells were irradiated with the indicated doses of IR and 72h later cell viability was evaluated by annexinV/PI staining. Data are the mean ± SD of three independent experiments (** p<0.01, * p<0.05 in OPM2, JJN3, RPMI-8226 and MM1S compared to LINF cells). (F) Increase in the percentage of cell death compared to untreated samples after the indicated treatments. Asterisks in samples treated with caffeine indicate significant values related to irradiated cells (** p<0.01, * p<0.05).

Cell cycle checkpoints are activated following induction of DSBs to provide time for the removal of the damage. The G2/M checkpoint has been described to efficiently retain cells in G2 until they contain less than 10–20 DSBs [26]. The fact that OPM2, JJN3, MM1S, and RPMI-8226 exhibited more γH2AX foci at 7 and 24h post-IR than the rest of the cell lines, prompted us to analyze the effect of ionizing radiation on cell cycle progression (Fig. 4A). We found that fast-growing HeLa and IM9 cells exhibited cell cycle arrest at 7h post-IR, but at 24h cells had repaired the lesions and escaped G2 arrest. Accordingly, HeLa and IM9 cell number increased 1.6±0.06 and 3.05±0.53-fold, respectively, at 24h post-IR compared to those present before irradiation. On the contrary, OPM2 and JJN3, with a doubling time similar to HeLa cells (indicated in Fig. 4A), also exhibited cell cycle arrest at 7h post-IR, but remained blocked at G2 phase at 24h post-IR and no increment in cell numbers were found 24h post-treatment (0.98±0.07 and 0.99±0.1-fold, respectively). U266, MM1S and RPMI-8226, were not arrested at 7h, probably due to their slower growth rate (doubling times from 30 to 39 hours). However, whereas MM1S and RPMI-8226 showed a clear G2 arrest at 24h post-IR and no increase in cell numbers before and 24h after irradiation were detected (0.8±0.25, and 1.1± 0.1-fold, respectively), U266 was not arrested in G2 at 24h, and a small increase in cell number was observed (1.3±0.2-fold). To further confirm that U266 was not arrested in G2 because most of the IR-induced lesions have been repaired (Fig. 2A to 2D), the level of damage was increased using 10 Gy of IR, and the cell cycle profile was analyzed at 24h post-IR (S1 Fig.). We observed that the population of U266 cells arrested in G2 at 24h post-IR also increased, indicating that this cell line is able to activate the DNA damage checkpoint. To confirm that G2 accumulation was due to prolonged checkpoint activation, cells were irradiated in the absence or in the presence of different checkpoint inhibitors. We used caffeine (4 mM), a well known inhibitor of DNA damage checkpoint sensor kinases ATM and ATR, UCN-01 (100 nM), which inhibits Chk1 (the downstream substrate of ATR) [27] and wortmannin (10 μM), which efficiently inhibits ATM and DNA-PK [28]. Treatment with caffeine had no effect on cell cycle distribution in U266, as expected, but efficiently abolished the G2 accumulation observed 24h post-IR in OPM2, JJN3 and MM1S (Fig. 4B), confirming that G2 accumulation was induced by checkpoint activation. Treatment with UCN-01 but not with wortmannin inhibited the G2 checkpoint in irradiated JJN3 cells, indicating that ATR was responsible for the G2 arrest in these cells. However, checkpoint activation seemed to depend on ATR but also on ATM/DNA-PK in OPM2 and MM1S (Fig. 4B), since treatment with UCN-01 and wortmannin abolished the G2 accumulation in these cell lines. It has been reported that high concentrations of caffeine could by itself induce G1 arrest [29]. To distinguish the effect of caffeine on abolishing G2/M checkpoint from its putative effect on G1 arrest, cells were irradiated with 2 Gy IR and 24h later, when MM cells were already blocked at G2, caffeine was added and maintained for a 6h period (Fig. 4C). We observed that treatment with the ATM/ATR inhibitor for 6h clearly diminished the amount of cells in G2 in OPM2, JJN3, MM1S and RPMI-8226, confirming that caffeine inhibited the G2 arrest. Importantly, we also observed that the 6h treatment resulted in dephosphorylation of γH2AX (Fig. 4D). This result indicated that the prolonged H2AX phosphorylation observed after irradiation depended on the presence of unrepaired DNA damage that signaled through the checkpoint kinases to H2AX, and not to defects in the dephosphorylation of the histone variant.

### Several MM cell lines are hypersensitive to IR and addicted to a functional G2 checkpoint for survival

As it has been described that cells retaining γH2AX foci at 24 h post-IR are more sensitive to IR [25,30,31,32], we further analyzed the survival of MM cell lines after different doses of IR by Annexin V/PI staining. The results showed that OPM2, JJN3, RPMI-8226 and MM1S were more sensitive to IR than U266 and H929 (p< 0.01) and control cell lines LINF167 and LINF692 (Fig. 4E).

We then reasoned that since the G2/M checkpoint response was stronger in some MM cell lines, these cells could exhibit a greater dependence on a functional checkpoint for survival to IR. To test this hypothesis, MM cell lines were irradiated, and 24h later, when repair-proficient cells should have repaired most of the DSBs, caffeine was added and maintained for a new 24h period. Treatment with the G2 checkpoint inhibitor for 24h increased cell death of irradiated JJN3, MM1S and RPMI-8226 cells, whereas it had no significant effect on H929, U266, or LINF control cells (Fig. 4F). These results confirmed our hypothesis and indicated that checkpoint responses are essential to protect some MM cell lines from cell death induced by DNA damage.

### Upregulation of proteins involved in DSB repair pathways in MM

**Fig 5:**
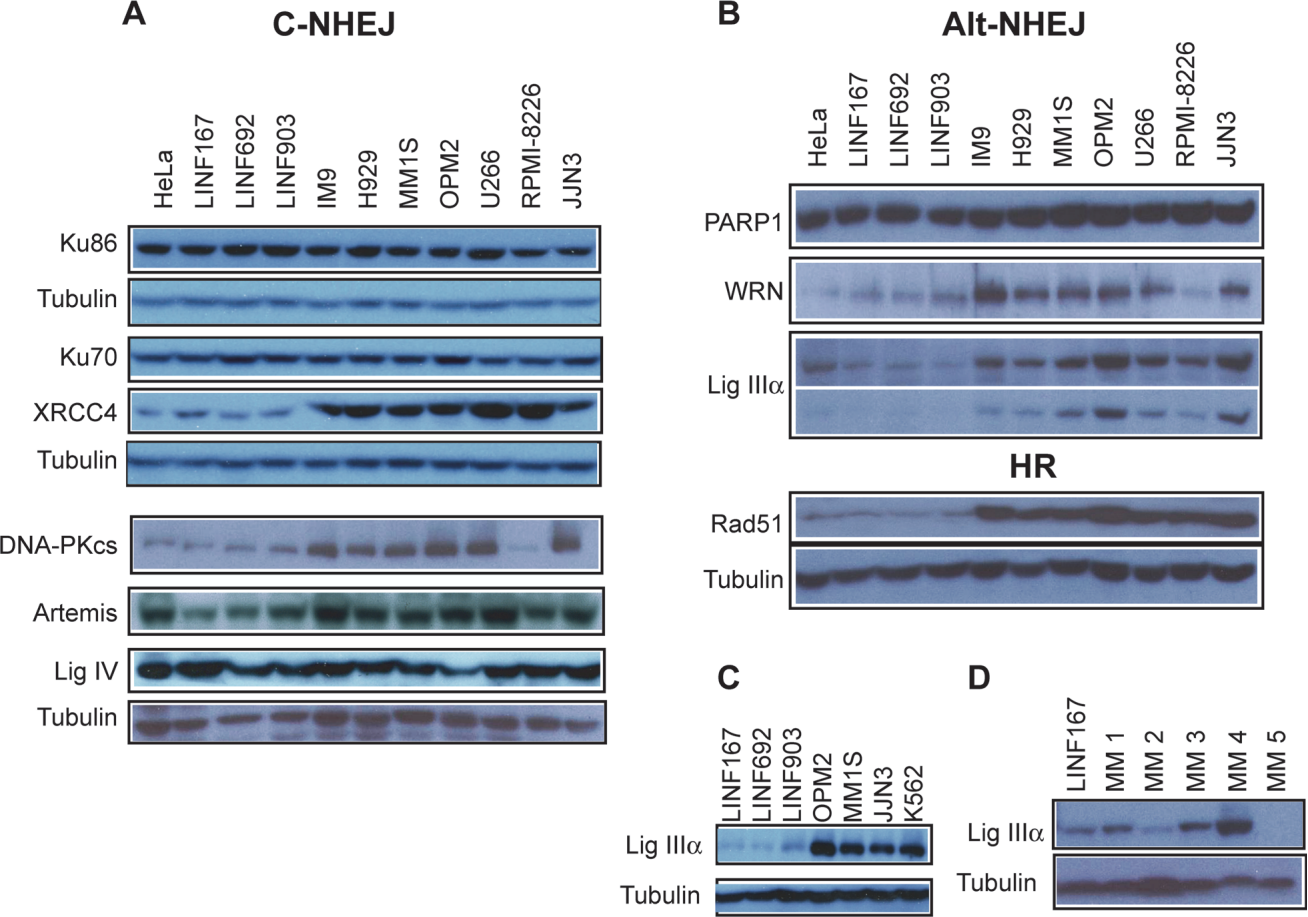
Western blot analysis of proteins involved in DSB repair in the indicated cell lines. (A) Proteins involved in the classical NHEJ pathway. (B) Levels of Alt-NHEJ proteins and the HR protein Rad51. (C) Levels of DNA ligase IIIα in LINF, MM and CML (K562) cell lines. (D) DNA ligase IIIα in plasma cell samples isolated from patients.

To further investigate DSB repair in MM cells we examined the steady-state levels of proteins involved in the two main pathways of DSB repair: NHEJ and HR. For this purpose, western blot analysis was performed in the 7 MM cell lines and compared with controls (3 LINF control cell lines and HeLa cells). No significant differences in protein levels of the key NHEJ proteins (Ku86, Ku70 and DNA ligase IV) were found among the samples (Fig. 5A). However, DNA-PKcs showed higher expression in 6 out of the 7 MM cell lines compared to controls. XRCC4 was clearly upregulated and Artemis was also increased in all MM cell lines compared to control cells (see quantifications in S2 Fig.).

Recent studies have shown the existence of an alternative NHEJ pathway (Alt-NHEJ) that mainly operates as a backup pathway [13]. Therefore, we analyzed the steady-state levels of several proteins involved in this pathway. Levels of PARP-1 were found similar in all the samples analyzed (Fig. 5B), whereas WRN protein was found upregulated in 6 out of the 7 MM cell lines. Of note, we found that all MM cell lines expressed higher levels of DNA ligase IIIα than controls, with MM1S, U266, JJN3 and specially OPM2 exhibiting the higher expression (Fig. 5B, see lower exposition and quantifications in S2 Fig.). Expression of DNA ligase IIIα in these cell lines was similar to that exhibited by K562, a chronic myeloid leukemia (CML) cell line previously shown to overexpress this protein [33] (Fig. 5C). Since DNA ligase IIIα has been extensively implicated in Alt-NHEJ [34,35,36,37], we decided to monitor the levels of this protein in plasma cells (PCs) isolated from patients with MM. We observed that the protein was upregulated in 3 out of the 5 samples analyzed, as compared with the linfoblastoid cell line, LINF167, used as control (Fig. 5D). Finally, we found that Rad51, a protein that plays an essential role exclusively in HR, was clearly upregulated in all MM cell lines (Fig. 5B).

### NHEJ efficiency is increased in MM cells

**Fig 6:**
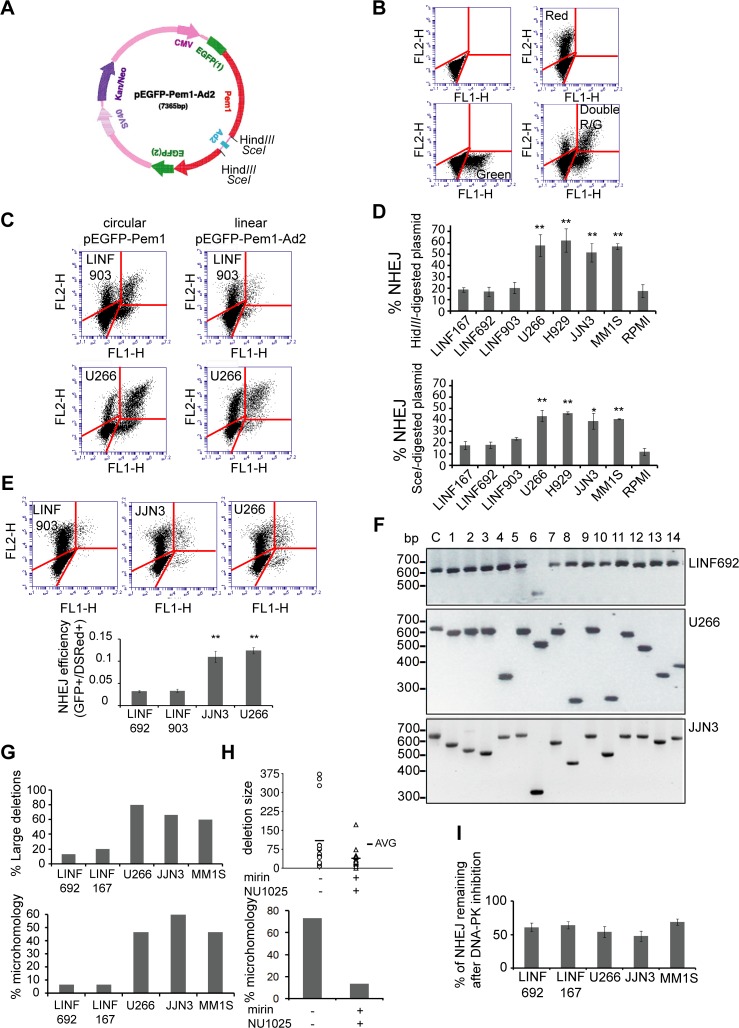
Analysis of NHEJ in MM cells. (A) Map of pEGFP-Pem1-Ad2 modified from reference [36]. (B) Dot plots of nontransfected LINF903 cells (panel 1), LINF903 cells transfected with 2 μg pDSRed2-N1 (panel 2), with 0.5 μg of pEGFP-Pem1 (panel 3), or with both plasmids together (panel 4). Numbers of green and red cells were determined 24h after transfection by FACS. (C) Dot plots of LINF903 and U266 cell lines transfected with 0.5 μg of pEGFP-Pem1 or 0.5 μg of *Hind*III-digested pEGFP-Pem1-Ad2 plasmid, together with 2 μg of pDSRed2-N1. Total represented events were adjusted to correct for differences in transfection efficiencies, and same numbers of cells transfected with circular and/or control pDSRed2-N1 are shown (6,000 cells). These numbers of events were then represented in panels corresponding to transfections with digested molecules. (D) Percentage of NHEJ of Hind*III*- or Sce*I*-digested plasmid in different cell lines. Mean of a minimum of three independent experiments is shown. (** p<0.01, * p<0.05, compared to LINF cells). (E) NHEJ efficiency in LINF, JJN3 and U266 cell lines carrying the integrated NHEJ reporter cassette. Stable pools were transfected with 5 μg of I-SceI endonuclease-expressing plasmid and 2 μg pDSRed2-N1 to correct for differences in transfection efficiencies. GFP+ and DsRed+ cells 24h after transfection are shown in panel 1 (A total of 6,000 GFP+ and/or red+ cells are shown). NHEJ efficiency was calculated as the ratio of GFP+/DsRed+ cells (panel 2). Data are the mean of three independent experiments (** p<0.01, compared to LINF cells). (F) Agarose gel showing PCR products of misrepaired products (1–14). The size of the PCR product from a correctly repaired DSB (c) is 628 bp. (G) Percentage of large deletions (≥20 bp, panel 1) and percentage of misrepaired plasmids using sequence microhomology (≥2 bp, panel 2) in LINF, U266, JJN3, and MM1S cell lines. (H) Deletion size (panel 1) and percentage of microhomology (panel 2) in plasmids recovered from U266 cells treated or not with mirin (100 μM) and NU1025 (50 μM). Cells were pretreated with the chemical inhibitors for 6h, transfected with *EcoR*I-digested plasmid, and cultured for 18h in the presence of the chemical inhibitors. (I) NHEJ activity remaining after inhibition of DNA-PKcs. Cells were transfected as in (C), and grown in the presence of 10 μM of NU7026.

To investigate the efficiency of NHEJ in MM we employed an extrachromosomal assay where end joining is determined by measuring the ability of the cells to recircularize an enzyme-digested plasmid (Fig. 6A). Plasmid recircularization results in the formation of the green fluorescent protein (GFP), and GFP+ cells can be easily detected and quantified by flow cytometry. Fig. 6B shows the fluorescence obtained by transfection of LINF903 cells with different controls. Dot plots of LINF903 and U266, representing cells transfected with the same amount of circular pEGFP-Pem1 or *Hind*III-digested pEGFP-Pem1-Ad2 plasmids, together with pDSRed2-N1, used to correct for transfection efficiency, are shown in Fig. 6C. We found that the number of GFP+ cells obtained by transformation with the linear, *Hind*III-digested, plasmid was higher in U266 than in LINF903 control cells, (Fig. 6C). In fact, frequency of NHEJ of *Hind*III or *Sce*I-digested plasmids (calculated by dividing numbers of GFP+ cells obtained by religation of the linearized plasmid by numbers of GFP+ cells obtained by transformation with the undigested plasmid, after normalizing for transfection efficiency), was found higher in most of the MM cell lines compared with LINF control cells, revealing an overactivation of NHEJ repair in MM (Fig. 6D).

To corroborate these results obtained using episomal plasmids, we used an intrachromosomal substrate, NHEJ-C, that was integrated into the chromatin of U266, JJN3 and control LINF cell lines. DSBs were generated by transfection of the stable cell lines with a I-SceI endonuclease-expressing plasmid, and NHEJ efficiency was estimated 24h later as the ratio of GFP+/DsRed+ cells. We found that NHEJ efficiency was significantly higher in MM compared to control LINF cell lines (Fig. 6E).

### MM cells show increased DNA deletions and microhomology use at DNA junctions

To molecularly characterize end joining repair, we used another *in vivo* assay that allows the calculation of different repair parameters: misrepair frequency, deletion size and use of microhomology at the repair junction. These parameters, when increased, indicate a higher use of the Alt-NHEJ pathway [13]. The assay consists on the transfection of *EcoR*I-digested pUC18 plasmid into the cells, the subsequent recovery of recircularized pUC18 from them, and transformation of bacterial cells for plasmid amplification and analysis. Since Alt-NHEJ proteins were found upregulated in all MM cells, we selected for the analysis those with higher transfection efficiency, U266, JJN3, and MM1S. Lymphoblastoid cells were used as healthy controls, although their low transfection efficiency and high transfection-associated cell death made us perform 50 transfections to obtain enough number of bacterial colonies for the analysis. Frequency of misrepair, that is white colonies (incorrectly repaired) *vs* total colonies (blue [correctly repaired]+ white), was found similar in U266, JJN3, MM1S, LINF692 and LINF167 cells (10.9± 2.2, 9.75± 1.62, 8.6±1.5, 10.05± 1.9 and 9.3± 2.5, respectively, was the mean of three independent experiments). However, PCR analysis, and sequencing of plasmids obtained from 15 white colonies from U266, JJN3, MM1S and LINF cells showed a clear increase in the number of large deletions in MM cells lines compared to LINF controls (Fig. 6F and 6G). Moreover, whereas a small percentage of DSBs were repaired using DNA sequence microhomology in lymphoblastoid cells, more than 40% of the breaks were repaired by a microhomology-mediated mechanims in U266, JJN3 and MM1S cells (Fig. 6G, panel 2). Deletion size and microhomology lengh are detailed in Tables A-E in S1 File. These results suggest that a high percentage of DSBs in MM cells may be repaired by Alt-NHEJ pathways, resulting in abnormal and highly mutagenic repair characterized by large DNA deletions and the use of sequence microhomology. To further demonstrate that these features were due to a higher use of the Alt-NHEJ pathway in MM, repair junctions were sequenced after chemical inhibition of several proteins involved in the pathway. U266 cells were treated with mirin, an inhibitor of the Mre11-Rad50-Nbs1 complex required for DNA resection and involved in both HR and Alt-NHEJ [38,39], and with NU1025, an inhibitor of PARP-1, a protein that has also been involved in the Alt-NHEJ pathway [40]. Treatment of U266 cells with 100 μM mirin, the concentration described to inhibit the MRN exonuclease activity [38], and 50 μM NU1025 reduced misrepair frequency from 12.3± 1.2 in the absence of treatment, to 5.3± 1.1 in the presence of the inhibitors (mean of three independent experiments, p<0.01). Sequencing of 15 plasmids derived from white colonies indicated that the presence of the chemical inhibitors clearly decreased both deletion size and microhomology use (Fig. 6H, panels 1 and 2, and Tables F-G in S1 File). We could not perform the experiment in JJN3 and MM1S because treatment with mirin resulted in a high percentage of cell death in these cell lines (more than 80% of the cells died compared to 40% in U266). We hypothesize that cell death could be related to a stronger requirement in JJN3 and MM1S of the MRN complex to repair their higher levels of endogenous DSBs (Fig. 2A).

To determine whether the increased activity of the Alt-NHEJ pathway in MM cells could be responsible for the higher frequency of NHEJ detected in the plasmid reactivation assays (Fig. 6D), we tested the effect of classical NHEJ inhibition, by the use of the specific DNA-PK inhibitor NU7026, on the efficiency of NHEJ in U266, MM1S, JJN3 and LINF control cells. Although the percentage of NHEJ remaining after DNA-PK inhibition was high (around 50%, in agreement with a previous report using DNA-PK mutants [41]), no significant differences were observed between MM and control LINF cells (Fig. 6I). These results suggest that the increased total NHEJ efficiency detected in MM cell lines compared to controls (Fig. 6D) seems to depend on the overactivation of both classical and DNA-PK-independent (included Alt-NHEJ) DSB repair pathways.

### HR efficiency is increased in MM cells

**Fig 7:**
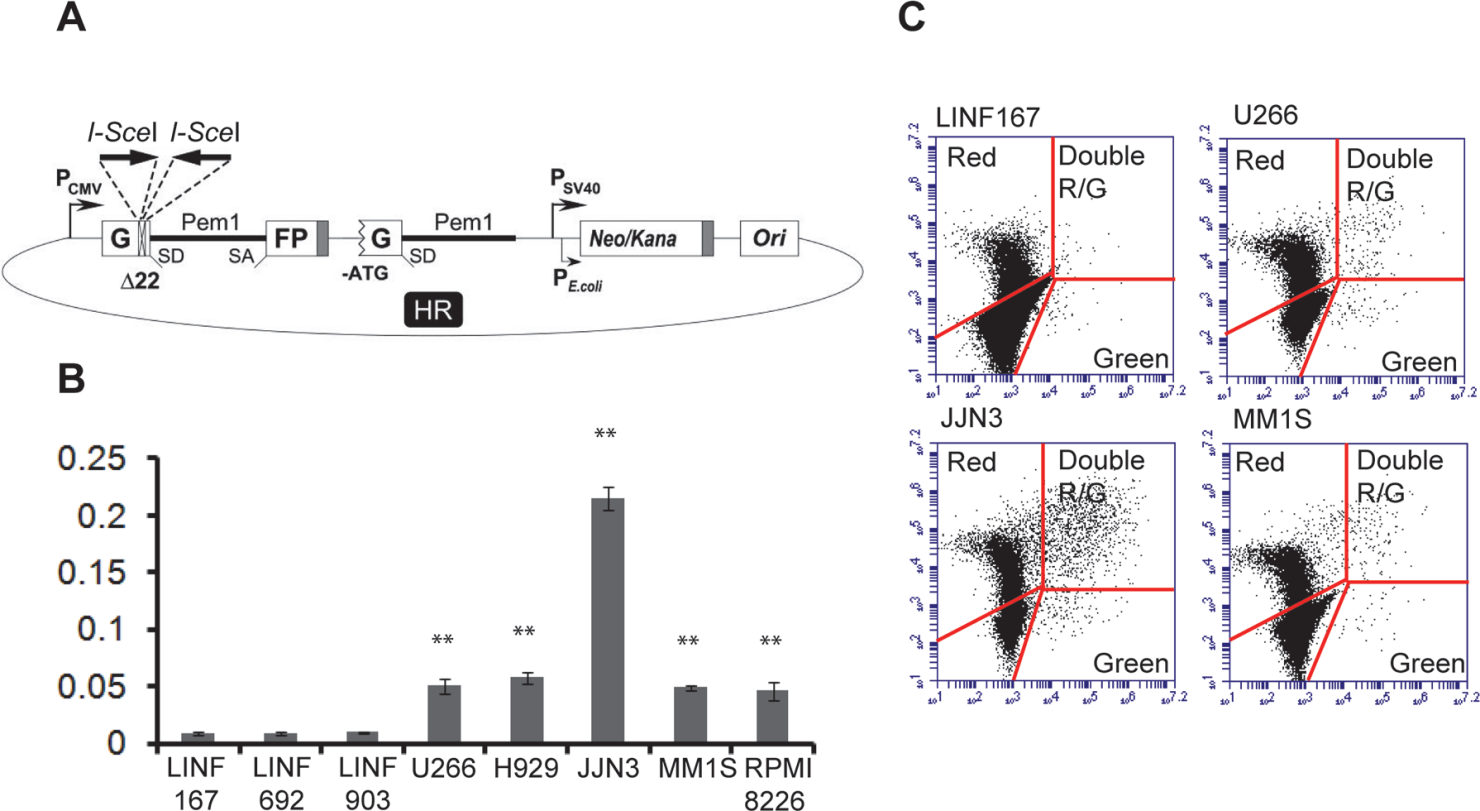
Analysis of HR in normal LINF and MM cell lines. (A) Reporter plasmid for detection of HR [22]. (B) Cells were transfected with 2 μg of *Sce*I-digested HR plasmid together with 2 μg of pDSRed2-N1 to normalize for the differences in transfection efficiency. Numbers of green and red cells were determined 48h after transfection by FACS. The ratio of GFP+ cells to DsRed+ cells was used as a measure of repair efficiency. Data are means ± SD of three independent experiments. (C) Representative images showing dot plots corresponding to the indicated cell lines. A total of 6,000 GFP+ and/or DsRed+ cells are shown. (** p<0.01, compared to LINF cells).

To analyze HR activity in MM we used the HR reported construct shown in Fig. 7A. The plasmid was linearized by digestion with *Sce*I and transfected into different MM and LINF cells lines. HR efficiency, calculated as the ratio of GFP+ cells to DsRed+, is shown in Fig. 7B. Interestingly, a significant increase of recombination activity was observed in all MM cell lines compared to control LINF cells (Fig. 7B and 7C).

## Discussion

DSBs are the most deleterious form of DNA damage; if left unrepaired they can cause cell death, if misrepaired, they lead to genomic instability and, ultimately, to the development or progression of cancer [42]. To handle this constant an inevitable threat, cells have developed several DSB repair pathways: HR, considered error free, although when constitutively activated can generate genomic rearrangements and lead to oncogenic activation [12], NHEJ, that can result in small insertions or deletions at the junction site, and Alt-NHEJ, a backup, highly mutagenic pathway that has been implicated in chromosomal translocation in mouse cells, [14]. In this study, we show that the three DSB repair pathways are upregulated in MM cells, both at the level of function and protein expression. This aberrant activation of DSB repair pathways, could contribute to the enormous genome instability found in MM.

Our initial experiments, measuring the repair kinetics of IR-induced DSBs by H2AX phosphorylation, suggested a defect in DSB repair in 4 out of 7 MM cell lines analyzed (Figs. 1 and 2). In agreement with our results, persistence of γ-H2AX foci 24h following irradiation has previously been reported for the RPMI-8226 MM cell line [43]. However, the neutral comet assay did not detect differences in repair kinetics between MM and normal control lymphoblastoid cells, which strongly suggests that MM cells are able to repair the majority of the breaks. We speculate that the higher percentage of large, and highly brilliant, γH2AX foci detected at long times after IR in OPM2, JJN3, MM1S and RPMI-8226, may correspond to persistent DSBs that could be under the limit of detection of the neutral comet assay (on the order of 50–75 breaks, as previously described [25]). In fact, most of the residual γH2AX foci were colocalized with the recombinase Rad51, which has also been found in association with persistent DSBs [44]. The subset of DSBs observed in these cell lines could represent lesions especially difficult to repair because of their complexity or to local chromatin organization.

Additional evidence for the presence of higher numbers of persistent DSBs in some MM cell lines came from the analysis of the cell cycle after treatment with IR. It has been described that duration of IR-induced G2/M cell cycle arrest depends on the level of damage and repair capacity. Thus, cells exposed to low levels of IR (below 2 Gy) usually do not show G2 arrest at 24h post-IR, whereas cells exposed to higher dose (10 Gy) show a clear cell cycle arrest [25]. On the other hand, it has been shown that checkpoint release occurs later in cells that accumulate DSBs, like those with defects in DNA repair, even when low doses of IR are applied [45]. Here, we show that the four MM cell lines exhibiting residual γH2AX foci also displayed a prolonged G2/M checkpoint activation 24h after 2 Gy. A plausible interpretation of these results is that the prolonged G2/M checkpoint activation arises from the presence of a subset of unrepaired DSBs in numbers enough to induce a persistent arrest. However, some defect in the checkpoint response cannot be ruled out. It is possible that MM1S and RPMI8226 cell lines present a G1 checkpoint deficiency compared to the control lines (Fig. 4A, 7H post-IR), which may lead to a replication stress and the appearance of spontaneous γH2AX foci.

Previous reports have shown that cell lines that retain higher numbers of γ-H2AX or Rad51 foci 24h post-IR are more sensitive to IR [25,30,31] and that persistent or irreparable DSBs induce cell death [32]. Therefore, the higher percentage of cells exhibiting H2AX foci 24h post-IR in OPM2, JJN3, MM1S and RPMI-8226, compared to the rest of the cell lines, possibly underlies their radiosensitive phenotype. We also propose that the prolonged G2/M arrest exhibited by these MM cell lines after irradiation is important for their survival, as shown by the increase in cell death after treatment with the checkpoint inhibitor caffeine.

The *in vivo* NHEJ functional assays indicate the absence of general DSB repair defects in MM. Moreover, 4 out of 5 MM cell lines analyzed exhibited an elevated NHEJ activity compared with normal lymphoblastoid cells (Fig. 6D). The analysis of proteins involved in NHEJ revealed no changes in the levels of Ku70 or Ku86 compared to controls. However, an upregulation of DNA-PKcs, Artemis and XRCC4 was found. Interestingly, higher expression of *XRCC4* has previously been reported in tumor samples isolated from patients with MM [46]. The upregulation of these NHEJ proteins is likely to contribute to the increased repair efficiency observed in MM cells. On this regard, a 4-fold increase in DNA-PKcs, and higher levels of NHEJ have also been described in CML compared to normal cells [11]. Moreover, high DNA-PKcs levels in chronic lymphocytic leukemia have been associated with poor prognosis [47]. NHEJ is not intrinsically inaccurate, but is versatile and adaptable to imperfect ends, which may result in short deletions or insertions [7]. Therefore, overactivity of this pathway may generate mutations, or even alignment of noncontiguous broken DNA ends, leading to translocations and deletions [11]. Overactivation of the NHEJ pathway would be especially dangerous in the presence of high levels of DNA damage.

Deregulation of the HR pathway also contributes to genome instability [10,12]. Thus, overexpression of Rad51 directly induces genome instability in the form of deletions, aneuploidy and multiple chromosomal rearrangements [48,49]. In CML, overactivation of the HR pathway has been described [50]. In MM, increased levels of *RAD51* and related genes, concomitant with an upregulated HR activity have previously been reported [51]. Our results confirm the increased levels of the recombinase Rad51 in all MM cell lines tested. Moreover, using a completely different HR functional assay, we show that HR activity is elevated in MM cells compared to normal lymphoblastoid controls.

Here, we describe for the first time, that MM cells also show elevated levels of proteins involved in Alt-NHEJ and an increased activity of this pathway, revealed by larger DNA deletions and higher microhomology use at repair junctions than control cells, that were reduced by chemical inhibition of the pathway. Moreover, upregulation of the Alt-NHEJ protein DNA ligase IIIα was also observed in plasma cells isolated from patients with MM. Interestingly, increased levels of DNA ligase IIIα have also been described in acute myeloid leukemia (AML) and CML, and a connection between increased Alt-NHEJ pathway and genome instability that drives disease progression has been proposed [33,52]. Levels of DNA ligase IIIα in MM cell lines were found to be similar to those exhibited by the CML cell line K562 (Fig. 5C). Although the rationale for altered levels of DNA ligase IIIα in CML or AML is not clear, it seems related to the constitutively activated kinase activities, and with lowered levels of some proteins involved in the canonical NHEJ [33,52]. However, this aspect remains controversial, since high levels of some proteins involved in classical NHEJ, together with increased NHEJ efficiency has also been described in CML [11]. In MM, we found that proteins involved in NHEJ are either unchanged or upregulated, and the activity of NHEJ was also elevated, suggesting that other causes may be responsible for DNA ligase IIIα protein upregulation.

The most likely explanation for the increased activity/protein levels of the three DSB repair pathways in MM (HR, NHEJ and Alt-NHEJ), would be the high level of endogenous DNA damage described in MM cells [24]. However, we cannot rule out the impact of additional factors, commonly upregulated in MM that could affect the expression of proteins involved in DSB repair. Thus, c-MYC, is known to upregulate Rad51 [53], NFkB, has been shown to increase HR [54], and KRAS has recently been associated with increased DNA ligase IIIα expression and preferential use of microhomology for end joining [55]. The contribution of these individual factors to DSB repair in MM needs to be further investigated.

In summary, our results show that NHEJ, HR and Alt-NHEJ pathways are stimulated in MM, in agreement with several reports that previously analyzed DSB repair in other hematological malignancies. Overactivation of the three repair pathways, and a putative competitive imbalance between them, might result in the emergence of genetic changes leading to disease progression and acquisition of drug resistances. In addition, the data reported here may be exploited therapeutically [56]. Given that many MM cell lines rely on a functional damage checkpoint, and exhibit increased activity of repair pathways, a therapy with checkpoint inhibitors and/or targeting these pathways would probably benefit MM patients. In fact, inhibitors of PARP, DNA ligase IIIα, and checkpoint proteins have been developed and are being tested for cancer treatment [56,57]. Interestingly, a combination of PARP and DNA ligase IIIα inhibitors has been recently assayed *in vitro* for the treatment of CML with promising results [56].

## Supporting Information

S1 Fig Cell cycle phase distribution of U266 before treatment (-IR) and 24h post-irradiation (2 Gy or 10 Gy). Percentages of cells in the different phases of the cell cycle are indicated.
(TIF)

S2 Fig Quantification of proteins. Band intensities were quantified using ImageJ, normalized to tubuline and calculated relative to LINF167 control cells. Error bars, when indicated, represent the standard deviation. Data shown are representative of at least two independent experiments. (** p<0.01, * p<0.05, compared to LINF cells). (A) Proteins involved in the classical NHEJ pathway. (B) Levels of Alt-NHEJ proteins and the HR protein Rad51. (C) Levels of DNA ligase IIIα in LINF, MM and CML (K562) cell lines. (D) DNA ligase IIIα in plasma cell samples isolated from patients.
(TIF)

S1 File Tables A-G. Sequence analysis of misrepaired plasmids from LINF692, LINF167, U266, JJN3 and MM1S. *EcoR*I site (GAATTC) is located at position 450–455 of plasmid pUC18 (indicated in lower case). Original sequences flanking the junctions are indicated. Nucleotides in the original sequences that are physically present after repair are underlined. Bolded nucleotides indicate microhomologies. After ligation only one copy of the microhomology sequence is preserved. Sequences marked in grey indicate insertions. Table A. LINF692. Table B. LINF167. Table C. U266. Table D. JJN3. Table E. MM1S. Table F. U266 in the absence of Alt-NHEJ protein inhibition. Table G. U266 with Alt-NHEJ protein inhibition.
(DOCX)
