# Supplementary figures and images for: Deregulation of DNA Double-Strand Break Repair in Multiple Myeloma: Implications for Genome Stability
Source: PLoS One. 2015 Mar 19;10(3):e0121581. doi: 10.1371/journal.pone.0121581 (PMC4366222; doi:10.1371/journal.pone.0121581)

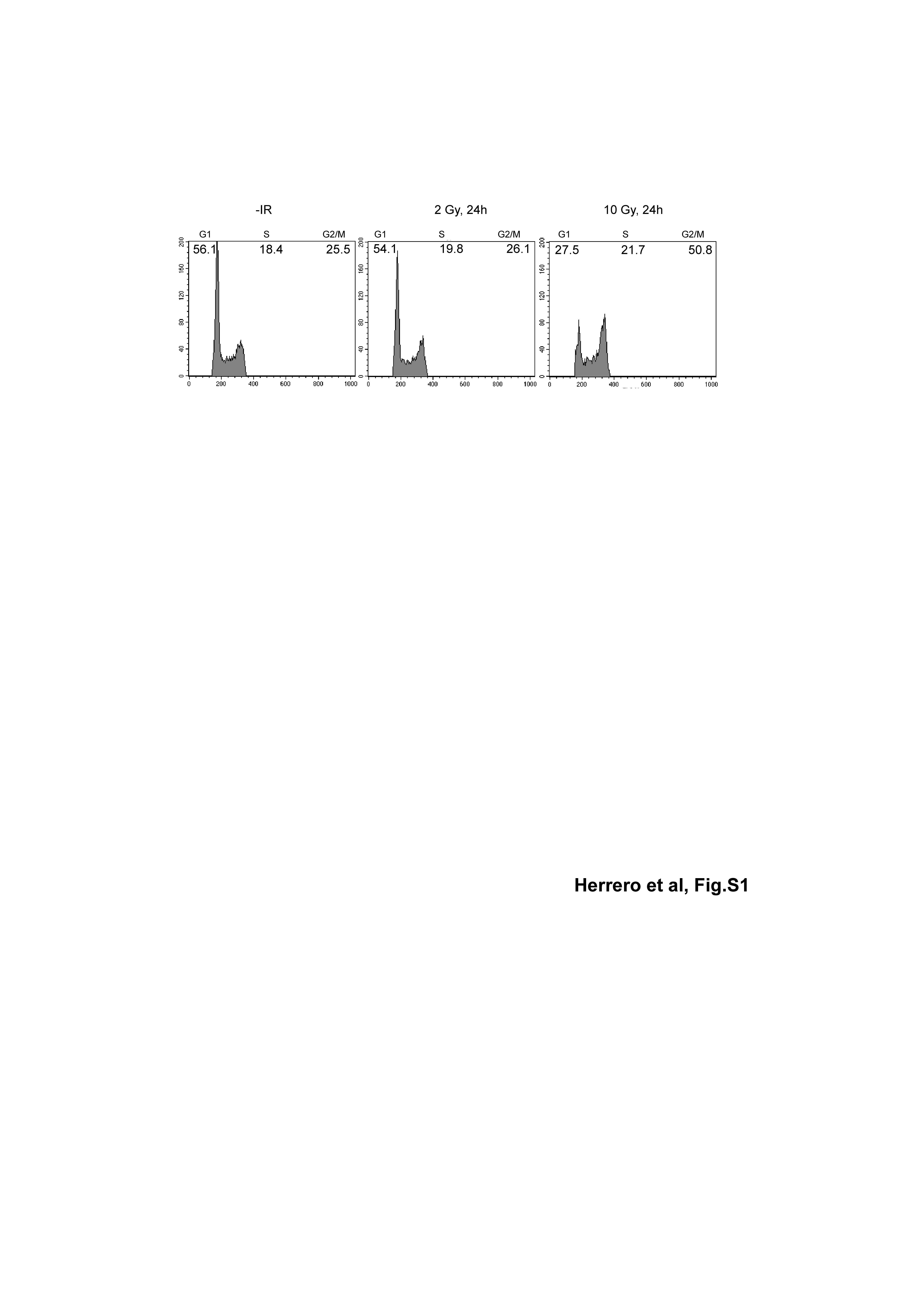

Supplement: S1 Fig — Percentages of cells in the different phases of the cell cycle are indicated. (TIF) [file pone.0121581.s001.tif]

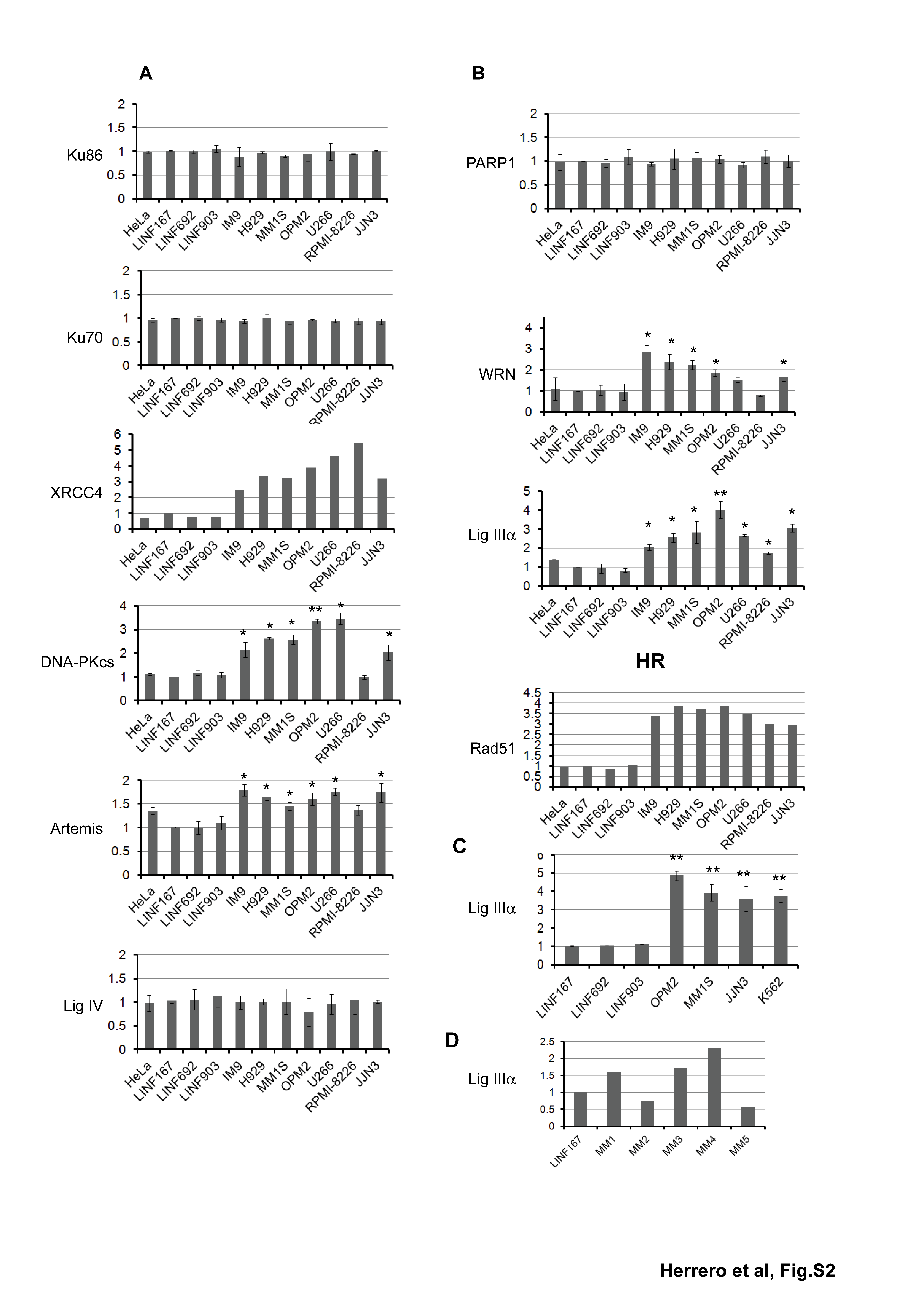

Supplement: S2 Fig — Band intensities were quantified using ImageJ, normalized to tubuline and calculated relative to LINF167 control cells. Error bars, when indicated, represent the standard deviation. Data shown are representative of at least two independent experiments. (** p<0.01, * p<0.05, compared to LINF cells). (A) Proteins involved in the classical NHEJ pathway. (B) Levels of Alt-NHEJ proteins and the HR protein Rad51. (C) Levels of DNA ligase IIIα in LINF, MM and CML (K562) cell lines. (D) DNA ligase IIIα in plasma cell samples isolated from patients. (TIF) [file pone.0121581.s002.tif]
