# Supplementary material for: Deregulation of DNA Double-Strand Break Repair in Multiple Myeloma: Implications for Genome Stability
Source: PLoS One. 2015 Mar 19;10(3):e0121581. doi: 10.1371/journal.pone.0121581 (PMC4366222; doi:10.1371/journal.pone.0121581)
Supplement: S1 File — Sequence analysis of misrepaired plasmids from LINF692, LINF167, U266, JJN3 and MM1S. EcoRI site (GAATTC) is located at position 450–455 of plasmid pUC18 (indicated in lower case). Original sequences flanking the junctions are indicated. Nucleotides in the original sequences that are physically present after repair are underlined. Bolded nucleotides indicate microhomologies. After ligation only one copy of the microhomology sequence is preserved. Sequences marked in grey indicate insertions. Table A. LINF692. Table B. LINF167. Table C. U266. Table D. JJN3. Table E. MM1S. Table F. U266 in the absence of Alt-NHEJ protein inhibition. Table G. U266 with Alt-NHEJ protein inhibition. (DOCX) [file pone.0121581.s003.docx]

**Tables**

**Tables A to G.** **Sequence analysis of misrepaired plasmids from LINF692, LINF167, U266, JJN3 and MM1S**. *EcoR*I site (GAATTC) is located at position 450-455 of plasmid pUC18 (indicated in lower case). Original sequences flanking the junctions are indicated. Nucleotides in the original sequences that are physically present after repair are underlined. Bolded nucleotides indicate microhomologies. After ligation only one copy of the microhomology sequence is preserved. Sequences marked in grey indicate insertions.

| Bacterial clone | Original sequences flanking breakpoint junctions  → Sequences at the junction | Deleted region (nt) | Bp deleted | Microhomology  length (bp) |
| --- | --- | --- | --- | --- |
| Blue control | GAGCTCgaattcGTAA | 0 | 0 | 0 |
| LINF692.1 | GGG**T**…AA**T**CATGGTC → GGG**T**CATGGTC | 441-461 | 20 | 1 |
| LINF692.2 | GAGCTCGAATTCGTAA → GAGCCGTAA | 448-454 | 7 | 0 |
| LINF692.3 | CTCGAATTCG → CTCGATTCG | 452-452 | 1 | 0 |
| LINF692.4 | CTCGAATTCGTA → CTCGAATCGTA | 454-454 | 1 | 0 |
| LINF692.5 | CTCGAATTCGTA → CTCGAATTGTA | 455-455 | 1 | 0 |
| LINF692.6 | GCAG**G**…CT**G**TCGTGC → GCAG**G**TCGTGC | 418-621 | 204 | 1 |
| LINF692.7 | GAATTCGTA → GAATCGTA | 454-454 | 1 | 0 |
| LINF692.8 | GAATTCGTA → GAATCGTA | 454-454 | 1 | 0 |
| LINF692.9 | GAGCTCGAATTCGTAA → GAGCCGTAA | 448-454 | 7 | 0 |
| LINF692.10 | GCTCGAATTCGTA → GCTCGCGTA | 451-454 | 4 | 0 |
| LINF692.11 | GAGCTCGAATTCGTAA → GAGCCGTAA | 448-454 | 7 | 0 |
| LINF692.12 | CGG**GTA**…**GTA**ATCATG→ CGG**GTA**ATCATG | 442-458 | 17 | 3 |
| LINF692.13 | GCTCGAATTCGTA → GCTCTCGTA | 450-453 | 4 | 0 |
| LINF692.14 | GAGCTCGAATTCGTAA → GAGCCGTAA | 448-454 | 7 | 0 |
| LINF692.15 | GCTCGAATTCGTA → GCTCGCGTA | 451-454 | 4 | 0 |
| Mean |  |  | 19.06 | 0.33 |

**Table A.** **LINF692.**

**Table B. LINF167.**

| Bacterial clone | Original sequences flanking breakpoint junctions  → Sequences at the junction | Deleted region (nt) | Bp deleted | Microhomology  length (bp) |
| --- | --- | --- | --- | --- |
| LINF167.1 | TACCGAGCTCGAATTCGTAAT → TACCGCGTAAT | 445-454 | 10 | 0 |
| LINF167.2 | GCCT**G**…TTC**G**TAATCATG → GCCT**G**TAATCATG | 414-456 | 43 | 1 |
| LINF167.3 | CTCGAATTCGTAATC → CTCGATCGTAATC | 452-453 | 2 | 0 |
| LINF167.4 | CTCGAATTCGTAAT → CTCGCGTAAT | 451-454 | 4 | 0 |
| LINF167.5 | CTCGAATTCGTAAT → CTCGCGTAAT | 451-454 | 4 | 0 |
| LINF167.6 | TACCGAGCTCGAATTCGT → TACCGCGT | 445-454 | 10 | 0 |
| LINF167.7 | TACCGAGC…TGGTCATAGCT → TACCGAGTCATAGCT | 446-464 | 19 | 0 |
| LINF167.8 | CTCGAATTCGTAATCAT → TACCGAGTCATAGCT | 454-457 | 4 | 0 |
| LINF167.9 | GGTCGA…CATGGTCATA → GGTCGAGGTCATA | 422-464 | 43 | 0 |
| LINF167.10 | GCTCGAATTCGTA → GCTCTCGTA | 450-453 | 4 | 0 |
| LINF167.11 | GGGTA**C**…GT**C**ATAGCT → GGGTA**C**ATAGCT | 443-467 | 25 | 1 |
| LINF167.12 | CTCGAATTCGTA → CTCGAATTGTA | 455-455 | 1 | 0 |
| LINF167.13 | CTCG**AAT**TCGT**AAT**CATGG → CTCG**AAT**CATGG | 454-460 | 7 | 3 |
| LINF167.14 | GAGCTCGAATTCGTAA → GAGCCGTAA | 448-454 | 7 | 0 |
| LINF167.15 | AGCTCGAATTCGTAATCAT → AGCTCGCGTAATCAT | 451-454 | 4 | 0 |
| Mean |  |  | 12.4 | 0.33 |

**Table C. U266.**

| Bacterial clone | Original sequences flanking breakpoint junctions  → Sequences at the junction | Deleted region (nt) | Bp deleted | Microhomology  length (bp) |
| --- | --- | --- | --- | --- |
| U266.1 | GGGTA**C**…GT**C**ATAGCT → GGGTA**C**ATAGCT | 443-467 | 25 | 1 |
| U266.2 | TACCGAGCT…TAATCATGG→ TACCGATCATGG | 446-459 | 14 | 0 |
| U266.3 | GAGCTCGA…ATGGTCATA → GAGCTGTCATA | 449-464 | 16 | 0 |
| U266.4 | GG**TAA**C**G**C…**TAA**T**G**AATCGGCC→ GG**TAA**C**G**CATCGGCC | 355-643 | 289 | 4 |
| U266.5 | TCGAT**T**TCG…GC**T**GTTTCC→ TCGAT**T**GTTTCC | 454-473 | 20 | 1 |
| U266.6 | CG**ATT**AA**GT**…CGA**ATT**C**GT**AATC→ CG**ATT**AA**GT**AATC | 345-457 | 113 | 5 |
| U266.7 | CCCGGGTAC…ATCATGGT→ CCCGGGATGGT | 340-461 | 22 | 0 |
| U266.8 | GGAA**GGG**C**GA**T…CGC**GGGGA**GAG→GGC**GGGGA**GAG | 271-655 | 385 | 5 |
| U266.9 | GTACCGAGCTCGAATTCGTA→ GTACCAATTCGTA | 444-450 | 7 | 0 |
| U266.10 | C**GCCAGCTG**…GT**GCCAGCTG**CAT→ C**GCCAGCTG**CAT | 312-633 | 322 | 8 |
| U266.11 | GCAGG**TCG**…AT**TCG**TAAACATG→ GCAGG**TCG**TAAACATG | 421-456 | 36 | 3 |
| U266.12 | T**GC**AG**G**…A**GC**CT**G**GGGTG→ T**GC**AG**G**GGGTG | 418-548 | 131 | 3 |
| U266.13 | A**TACCGC**A**CA**…GT**TA**T**CCGC**T**CA**CA→ A**TACCGC**A**CA**CA | 208-502 | 295 | 8 |
| U266.14 | CGAATTCGT…CGCTCTTCC→ CGAATTTCTTCC | 455-684 | 230 | 0 |
| U266.15 | TCGAAT**T**…CA**T**ACGAGCC→ TCGAAT**T**ACGAGCC | 455-518 | 64 | 1 |
| Mean |  |  | 131.2 | 2.6 |

**Table D. JJN3.**

| Bacterial clone | Original sequences flanking breakpoint junctions  → Sequences at the junction | Deleted region (nt) | Bp deleted | Microhomology  length (bp) |
| --- | --- | --- | --- | --- |
| JJN3.1 | CGACGGCC**AG**…**AG**CTGTTTC→ CGACGGCC**AG**CTGTTTC | 395-471 | 77 | 2 |
| JJN3.2 | CGACGT**…** TCACAATTCC→ CGACGT CCTCACAATTCC | 378-499  +2bp insertion (grey) | 122 | 0 |
| JJN3.3 | GTC**GA**CT**CTA**…**GA**G**CTA**ACTC→ GTC**GA**CT**CTA**ACTC | 426-569 | 144 | 5 |
| JJN3.4 | GA**G**C**TC**G**A**…**G**G**TCA**TAGCT → GA**G**C**TC**G**A**TAGCT | 452-468 | 17 | 4 |
| JJN3.5 | CTCGAATTCGTAAT → CTCGAATTAATTCGTAAT | +4 insertion (grey) | +4 | 0 |
| JJN3.6 | **TGC**G**TAA(**G)**GAG**...**TGC**C**TAA**T**GAG**TG→**TGCTAA**T**GAG**TG | 219-563 | 345 | 9(G at position 213 is deleted, G at position 217 is mutated to T |
| JJN3.7 | CTAGAG**G**A**TC**…**G**C**TC**ACAATT→ CTAGAG**G**A**TC**ACAATT | 434-501 | 68 | 3 |
| JJN3.8 | ACGTTGTAA**A**…**A**CTGCC→ ACGTTGTAA**A**CTGCC | 384-594 | 211 | 1 |
| JJN3.9 | ACCG**AGCT**…**AGCT**GTTT→ ACCG**AGCT**GTTT | 449-473 | 20 | 4 |
| JJN3.10 | G**TC**G**AC**TC**TA**…C**TCAC**AT**TA**ATTG→ G**TC**G**AC**TC**TA**TTG | 427-580 | 154 | 6 |
| JJN3.11 | GCTCGA**AT**TCGT**AT**CATGGT → GCTCGA**AT**CATGGT | 454-459 | 6 | 2 |
| JJN3.12 | CTCGAA**T**TCG**T**AATCATGG→ CTCGAA**T**AATCATGG | 454-457 | 4 | 1 |
| JJN3.13 | CTCG**AATT**C…**AATT**GTTATC→ CTCGTTATC | 451-491 | 41 | 4 |
| JJN3.14 | CCGGGTAC…GAATTCGT→ CCGGGTTCGT | 440-452 | 13 | 0 |
| JJN3.15 | CCCGGG**T**A**C**…**T**C**C**TGTGTG→ CCCGGG**T**A**C**TGTGTG | 443-479 | 37 | 2 |
| Mean |  |  | 84.2 | 2.86 |

**Table E. MM1S.**

| Bacterial clone | Original sequences flanking breakpoint junctions  → Sequences at the junction | Deleted region (nt) | Bp deleted | Microhomology  length (bp) |
| --- | --- | --- | --- | --- |
| MM1S.1 | GGG**T**A**CC**…AA**T**T**CC**ACACAA→ GGG**T**A**CC**ACACAA | 444-509 | 10 | 3 |
| MM1S.2 | GGGTA**C**…AAT**C**ATGGTCAT→ GGGTA**C**ATGGTCAT | 443-461 | 19 | 1 |
| MM1S.3 | GGATCC…AATTCGTAAT→ GGATCCTCGTAAT | 435-453 | 19 | 0 |
| MM1S.4 | CG**GAATT**C**GT**A**ATC**ATGG**TCA**T**A**GC…**GAA**A**TTGT**T**ATC**CGC**TCA**C**A**AT→CG **GAA**A**TTGT**T**ATC**CGC**TCA**C**A**AT | 450-484 | 34 | 14 |
| MM1S.5 | GGGTA**C**…**C**ATGGTCATAGCT→ GGGTA**C**ATGGTCATAGCT | 443-461 | 19 | 1 |
| MM1S.6 | TT**ATCA**G**GGT**T**AT**T**G**…**ATCA**T**GGT**C**AT**A**G**CT→ TT**ATCA**G**GGT**T**AT**T**GATCA**T**GGT**C**AT**A**G**CT | 2519-458 | 626 | 10 |
| MM1S.7 | **GACC**A**TG**ATTA**C**…**GACCTGC**AGGCATGC→ **GACC**A**TG**ATTAAGGCATGC | 450-491 | 43 | 7 |
| MM1S.8 | AGCT**AT**…**AT**TCGAG→ AGCT**AT**TCGAG | 438-453 | 16 | 2 |
| MM1S.9 | TTTCACACA…GAATTCGAG→ TTTCACTTCGAG | 423-452 | 30 | 0 |
| MM1S.10 | A**TCC**C**CGGG**TAC…T**TCC**AGT**CGGG**AAACCT→ A**TCC**C**CGGG**TACAAACCT | 443-614 | 172 | 7 |
| MM1S.11 | GGAACAGC…CGAGCTCGGT→ GGAACGCTCGGT | 432-457 | 26 | 0 |
| MM1S.12 | TGCCTG**CA…CA**T(G)GTCATA→ TGCCTG**CA**TTGTCATA | 416-463 | 48 | 2 (G at position 464 is mutated to T) |
| MM1S.13 | TCGAATTCGTAATCATGGT→ TCGAATTGTAATCATGGT | 455-455 | 1 | 0 |
| MM1S.14 | GCC**AGTG**…**AG**C**TG**TTTCC→ GCC**AGTG**TTTCC | 397-474 | 78 | 4 |
| MM1S.15 | CCGGGTAC…ATCATGGTC→ CCGGGATGGTC | 440-461 | 22 | 0 |
| Mean |  |  | 77.5 | 3.4 |

**Table F. U266 in the absence of Alt-NHEJ protein inhibition.**

| Bacterial clone | Original sequences flanking breakpoint junctions  → Sequences at the junction | Deleted region (nt) | Bp deleted | Microhomology  length (bp) |
| --- | --- | --- | --- | --- |
| U266.1 | CG**AAT**TCGT**AAT**CATG→ CG**AAT**CATG | 454-460 | 7 | 3 |
| U266.2 | AGCT**T**GC…AAT**T**CGTAATC→ AGCT**T**CGTAATC | 405-454 | 50 | 1 |
| U266.3 | AGAGGA**TCC**…AA**TTC**ACA→ AGAGGA**TCC**ACA | 435-509 | 75 | 3 |
| U266.4 | CC**AGTG**CCA…T**AG**C**TG**TTTCC→ CC**AGTG**TTTCC | 397-474 | 78 | 4 |
| U266.5 | TG**CATG**…T**CATG**GTCATAG→ TG**CATG**TCATAG | 410-465 | 56 | 4 |
| U266.6 | GAATTCCGTAATCATG→ GAATTATG | 455-462 | 8 | 0 |
| U266.7 | GGA**TC**CC…**TC**CTGTGT→ GGA**TC**TGTGT | 434-479 | 46 | 2 |
| U266.8 | A**G**T**T**G**GGT**AA…C**G**C**T**C**GGT**CGTTC→ A**G**T**T**G**GGT**CGTTC | 350-722 | 373 | 5 |
| U266.9 | TCGAAT…TAGCTGTT→ TCGAATGCTGTT | 454-470 | 17 | 0 |
| U266.10 | T**A**G**A**G**GA**TC…**A**T**A**C**GA**GCCGGA→ T**A**G**A**G**GA**GCCGGA | 432-614 | 91 | 4 |
| U266.11 | TA**C**T**GA**G**AG**T…GC**C**G**GAAG**CATAAAGT→ TA**C**T**GA**G**AG**TATAAAGT | 178-531 | 354 | 5 |
| U266.12 | T**A**G**A**G**GA**TC**CC**CG**…**C**A**T**A**C**GA**G**CC**GGA→ T**A**G**A**G**GA**TC**CC**GGA | 436-525 | 90 | 6 |
| U266.13 | T**CTTC**GCTA… C**CT**G**TC**GTGC→C**CT**G**TC**TTTTGTGC | 297-623 | 327 | 4 |
| U266.14 | TCC**CC**GGG…T**CC**TGTGT→ TCC**CC**TGTGT | 437-479 | 43 | 2 |
| U266.15 | CCGGGTA…TCATGGTC→ CCGGGATGGTC | 440-461 | 22 | 0 |
| Mean |  |  | 109.13 | 2.86 |

**Table G. U266 with Alt-NHEJ protein inhibition.**

| Bacterial clone | Original sequences flanking breakpoint junctions  → Sequences at the junction | Deleted region (nt) | Bp deleted | Microhomology  length (bp) |
| --- | --- | --- | --- | --- |
| U266.1 | tctagggg…AATCATG→ tctagGTCATG | 429-459 | 31 | 0 |
| U266.2 | GGGTA**C**…GT**C**ATAGC → GGGTACATAGC | 442-467 | 25 | 1 |
| U266.3 | TCGAAT**T**CG…A**T**AGCTGT→ TCGAAT**T**AGCTGT | 455-469 | 15 | 1 |
| U266.4 | CGCGGAC…ATTGTTATC→ CGCGGGTTATC | 441-490 | 50 | 0 |
| U266.5 | CG**AGCT**CG…AT**AGCT**GTT→ CG**AGCT**GTT | 449-473 | 25 | 4 |
| U266.6 | GAATTCGTAATCATG→ GAATTAATTCGTAATCA | +4 insertion (grey) | +4 | 0 |
| U266.7 | TTGTAAAA…ATTCGTAAT→ TTGTACGTAAT | 382-454 | 73 | 0 |
| U266.8 | GATCCCC…AATTCGTAA→ GATCCTCGTAA | 435-453 | 19 | 0 |
| U266.9 | CGGG**T**ACC…C**T**GTTTCC→ CGGG**T**GTTTCC | 441-473 | 33 | 1 |
| U266.10 | GAT**CGG**TG…GC**CGG**AAGC→ GAT**CGG**AAGC | 283-454 | 172 | 0 |
| U266.11 | GCTCGAAT…GTTATCC→ GCTCGTATCC | 451-492 | 42 | 0 |
| U266.12 | GAA**TT**CGT…G**TT**TCC→ GAA**TT**TCC | 455-476 | 22 | 2 |
| U266.13 | CGAATTCG…ATCATG→ CGAATTATG | 455-461 | 7 | 0 |
| U266.14 | GAATTCG…TCCGCTG→ GAATTGCTG | 455-497 | 43 | 0 |
| U266.15 | GGGTA**C**…GT**C**ATAGC → GGGTACATAGC | 442-467 | 25 | 1 |
| Mean |  |  | 38.8 | 0.66 |
